# High-Level Gene Flow Restricts Genetic Differentiation in Dairy Cattle Populations in Thailand: Insights from Large-Scale Mt D-Loop Sequencing

**DOI:** 10.3390/ani11061680

**Published:** 2021-06-04

**Authors:** Nattakan Ariyaraphong, Nararat Laopichienpong, Worapong Singchat, Thitipong Panthum, Syed Farhan Ahmad, Danai Jattawa, Prateep Duengkae, Narongrit Muangmai, Thanathip Suwanasopee, Skorn Koonawootrittriron, Kornsorn Srikulnath

**Affiliations:** 1 Animal Genomics and Bioresource Research Center (AGB Research Center), Faculty of Science, Kasetsart University, 50 Ngamwongwan, Chatuchak, Bangkok 10900, Thailand; nattakan.ariya.58@gmail.com (N.A.); nararat.l@ku.th (N.L.); worapong.si@ku.th (W.S.); thitipong.pa@ku.th (T.P.); farhan.phd.unesp@gmail.com (S.F.A.); prateep.du@ku.ac.th (P.D.); 2 Laboratory of Animal Cytogenetics and Comparative Genomics (ACCG), Department of Genetics, Faculty of Science, Kasetsart University, Bangkok 10900, Thailand; 3 Special Research Unit for Wildlife Genomics (SRUWG), Department of Forest Biology, Faculty of Forestry, Kasetsart University, 50 Ngamwongwan, Chatuchak, Bangkok 10900, Thailand; 4 Tropical Animal Genetic Special Research Unit, Department of Animal Science, Faculty of Agriculture, Kasetsart University, Bangkok 10900, Thailand; fagrdnj@ku.ac.th (D.J.); agrtts@ku.ac.th (T.S.); 5 Department of Fishery Biology, Faculty of Fisheries, Kasetsart University, Bangkok 10900, Thailand; seaweed_53@hotmail.com; 6 Center of Excellence on Agricultural Biotechnology (AG-BIO/MHESI), Bangkok 10900, Thailand

**Keywords:** D-loop, dairy cattle, *Bos taurus*, *Bos indicus*, genetic diversity

## Abstract

**Simple Summary:**

Domestication and artificial selection lead to the development of genetically divergent cattle breeds or hybrids that exhibit specific patterns of genetic diversity and population structure. Development of mitochondrial markers has allowed investigation of cattle diversity worldwide; however, an extensive study on the population-level genetic diversity and demography of dairy cattle in Thailand is still needed. Reduction in the genetic diversity of livestock also decreases the species resilience and adaptability to local environmental conditions and disease outbreaks, thus leading us to hypothesize that Thai dairy cattle populations are approaching a status of low genetic variability. In the present study, the genetic diversity and structure of Thai dairy cattle populations were investigated, both within and between populations, for 179 individuals from nine provinces of Thailand. Mitochondrial D-loop sequence data were collected and analyzed. To minimize the degree of relationship among individuals, cattle were randomly selected based on details interviewed with the owners. The results will facilitate an improved understanding of the fundamental elements involved in breeding strategies and promote sustainable livestock utilization.

**Abstract:**

Domestication and artificial selection lead to the development of genetically divergent cattle breeds or hybrids that exhibit specific patterns of genetic diversity and population structure. Recently developed mitochondrial markers have allowed investigation of cattle diversity worldwide; however, an extensive study on the population-level genetic diversity and demography of dairy cattle in Thailand is still needed. Mitochondrial D-loop sequences were obtained from 179 individuals (hybrids of *Bos taurus* and *B. indicus*) sampled from nine different provinces. Fifty-one haplotypes, of which most were classified in haplogroup “I”, were found across all nine populations. All sampled populations showed severely reduced degrees of genetic differentiation, and low nucleotide diversity was observed in populations from central Thailand. Populations that originated from adjacent geographical areas tended to show high gene flow, as revealed by patterns of weak network structuring. Mismatch distribution analysis was suggestive of a stable population, with the recent occurrence of a slight expansion event. The results provide insights into the origins and the genetic relationships among local Thai cattle breeds and will be useful for guiding management of cattle breeding in Thailand.

## 1. Introduction

Cattle are among the most economically important domesticated animals in the world. The majority are humpless taurine (*Bos taurus*) [1] and zebu (*B. indicus*) [1] that likely originated from the aurochs (*B. primigenius*) through a domestication event some 8000–10,000 years ago, and then spread globally by means of human trade and migration [2,3]. *Bos taurus* produces high quality and yield of milk, especially the Holstein and European cattle breeds. By contrast, *B. indicus* has lower milk production but is more tolerant to high temperature and humidity [4]. The beneficial traits of both species have been utilized to improve genetic diversity through global programs of crossbreeding [5,6]. Thailand is a tropical country in Southeast Asia characterized by high temperature (32.7–34.9 °C) and high relative humidity (60–82%) [7]. Historically, cattle in Thailand were used primarily for draft and meat production [8], but in the 1950s cooperation between the Thai government and the United Nations led to the initiation of a milk program for schools to improve the nutritional status of local communities by providing powdered and fresh milk [9]. Purebred Holstein sires and dams were imported to improve milk quality and yield in livestock demonstration farms under sustainable management [5,10]. However, purebred Holstein cattle are unsuited to the Thai tropical environment; therefore, crossbreeding has occurred between various breeds of *B. indicus* (such as Red Sindhi, Sahiwal, Brahman, and Thai native) and *B. taurus* (such as Brown Swiss, Jersey, and Red Danish) [11]. Currently, 95% pure Holstein trait is retained in less than 75% of the Thai dairy cattle population, whereas other populations exhibit multi-breed traits [12,13,14]. This observation indicates that Thai dairy cattle have adapted to or undergone selection for the local environment [15].

The Dairy Farming Promotion Organization of Thailand (D.P.O.) has obtained data on economically important traits of individual dairy cattle that have complete breed-fractions in Thailand [16]. The central part of Thailand has weather suitable for dairy cattle farming and it was chosen to be the main place for the national dairy activities, training, and organization [17,18]. In 2008, the central region produced 1080 tons/d of milk (67% of national production). Thai dairy cattle comprise rich breeds with a long history in agriculture, but few records exist concerning their pedigree, mating selection, traits, farm management, and genetic diversity. Farmers rely on personal experience to make breeding decisions, hence genetic improvement and management of cattle are limited [19,20]. Commercial and industrialized livestock production systems have expanded throughout the country, and it is likely that increased use of highly productive commercial cattle has reduced genetic diversity within the population [21]. Reduction in the genetic diversity of livestock also decreases the species resilience and adaptability to local environmental conditions and disease outbreaks [22]. These findings led us to hypothesize that Thai dairy cattle populations are approaching a status of low genetic variability.

In assessments of genetic variation among dairy cattle in other Asian countries, various phylogenetically distinct clusters have been resolved [23]. The mitochondrial displacement-loop (mt D-loop) region is a powerful genetic marker that is useful for investigating origin, genetic diversity, and relationships among cattle breeds and species [24,25,26]. In the present study, the genetic diversity and structure of Thai dairy cattle populations were investigated, both within and between populations, for 179 individuals from nine provinces (Chiang Mai, Khon Kaen, Lopburi, Udon Thani, Saraburi, Ratchaburi, Prachuap Khiri Khan, Phetchaburi, and Nakhon Ratchasima), with the hub of dairy cattle farming in Thailand in Saraburi (D.P.O. recommendation). Mitochondrial D-loop sequence data were collected and analyzed. To minimize the degree of relationship among individuals, cattle were randomly selected based on details such as farm management and attitude for semen selection collected from interviews with the owners.

## 2. Materials and Methods

### 2.1. Specimen Collection and DNA Extraction

A total of 179 individuals cattle were sampled in nine provinces of Thailand. The number of samples from each farm depended on permission from the owners. Detailed information on the sampled populations is presented in Table 1. Blood specimens were collected from the jugular vein using a Vacuette^®^ 21-gauge needle containing 6 mL EDTA (Greiner Bio-One, Kremsmünster, Austria). Animal care and all experimental procedures were approved by the Animal Experiment Committee, Kasetsart University, Bangkok, Thailand (ACKU60-AGR-009) and conducted in accordance with the Regulations on Animal Experiments at Kasetsart University. Total genomic DNA was extracted using a MasterPure™ DNA Purification Kit (Epicentre^®^, Madison, WI, USA) following the manufacturer’s instructions. The DNA quality and concentration were determined using a spectrophotometer (NanoDrop™ 2000, Thermo Scientific, Wilmington, DE, USA).

### 2.2. Mitochondrial D-Loop Sequencing

The mt D-loop fragments were amplified following the method of [27] using the primers Mito (D-loop) F (5′-TAGTGCTAATACCAACGGCC-3′) and Mito (D-loop) R (5′-AGGCATTTTCAGTGCCTTGC-3′). Each PCR amplification was performed using 15 μL of 1 × ThermoPol^®^ buffer that contained 1.5 mM MgCl_2_, 0.2 mM dNTPs, 5.0 μM primers, 0.5 U *Taq* polymerase (Apsalagen Co., Ltd., Bangkok, Thailand), and 25 ng genomic DNA. The PCR conditions were as follows: initial denaturation at 94 °C for 3 min, followed by 35 cycles of 94 °C for 30 s, 52 °C for 40 s, and 72 °C for 1 min 30 s, and final extension at 72 °C for 10 min. The PCR products were detected by electrophoresis in 1% agarose gel. The PCR products were purified using the GenUP™ PCR Cleanup Kit (Biotechrabbit, Hennigsdorf, Germany). Nucleotide sequences of the DNA fragments were determined by the DNA sequencing service of First Base Laboratories Sdn Bhd (Seri Kembangan, Selangor, Malaysia). The BLASTn and BLASTx programs (http://blast.ncbi.nlm.nih.gov/Blast.cgi) (accessed on 18 April 2020). were used to search nucleotide sequences in the National Center for Biotechnology Information database to confirm the identity of the amplified DNA fragments. The sequences generated were deposited in the DNA Data Bank of Japan (DDBJ) (Table S1).

### 2.3. Sequence Analysis

Multiple sequence alignment was performed for 187 (179 + 8) sequences in the mt D-loop data set, including four sequences (GenBank accession numbers: FN5573888, KX770828, AB003799, and KR857571) of *B. taurus*, two sequences (GenBank accession numbers: EF524185 and KU682489) of *B. indicus*, and two sequences (GenBank accession numbers: KR008119 and KU687004) of *Bubalus bubalis* (Linnaeus, 1758) [1] retrieved from GenBank. The sequences were aligned using the default parameters of the Molecular Evolutionary Genetics Analysis X (MEGAX) software [28]. All unalignable and gap-containing sites were removed carefully and trimmed from the data sets. Estimates of haplotype (*h*) and nucleotide (*π*) diversity [29], number of haplotypes (*H*), the estimator theta (*S*), overall haplotype, and average number of nucleotide differences (*k*) were calculated based on the mt D-loop sequences, as implemented in DnaSP version 6 [30]. The genetic differentiation coefficient (*G*_ST_), Wright’s *F*-statistic for subpopulations within the total population (*F*_ST_), *Φ*_ST_ values, and gene flow (*N*_m_) from the sequence data and haplotype data were estimated using Arlequin version 3.5.2.2 [31]. The *F*_ST_ and *Φ*_ST_ values were calculated by analyzing 1000 permutations of haplotypes between populations [32]. The *F*_ST_ statistic is based only on the difference in haplotype frequencies, whereas *Φ*_ST_ considers the relationships between haplotypes based on the molecular genetic distance [33]. The average number of nucleotide substitutions per site between populations (*D*_xy_) and the net nucleotide substitutions per site between populations (*D*_a_) were assessed using DnaSP version 6 [30].

Evaluation of cluster membership and testing this as a potential consequence of isolation-by-distance is important to determine whether genetic differentiation increases with geographic distance [34,35]. The relationship between nucleotide divergence and geographic distance was assessed using a Mantel test [36], as implemented in Alleles In Space (AIS) [37], with 1000 permutations to establish the significance of the correlation coefficient (null hypothesis: genetic clustering is a result of isolation-by-distance, *p* < 0.01). To visualize spatial patterns of genetic diversity, a landscape shape interpolation (LSI) analysis was conducted, wherein geographic coordinates (*x*- and *y*-axes) were related to nucleotide diversities (surface plot heights, *z*-axis) in a three-dimensional surface plot. Peaks in the surface plot represent areas of high nucleotide diversity between individuals, so can be considered to be areas of restricted gene flow, whereas troughs in the surface plot reveal areas of low nucleotide diversity. The LSI analysis was performed with AIS software using a distance weighting parameter (α) of 1 and grid settings of 80 × 80. All analyses implemented in AIS used sequences as the input matrix (raw genetic distances) and Universal Transverse Mercator (UTM) coordinates. ArcGIS [38] was used to construct maps for each population data set and to incorporate information on cluster membership.

A statistical parsimony network of the consensus sequences was constructed using the Templeton, Crandall, and Sing (TCS) algorithm implemented in PopART version 1.7 to examine haplotype grouping and population dynamics [39]. Demographic history was determined using the statistical test of neutrality. Tajima’s *D* [40], Fu and Li’s *D* * and *F* * tests [41], Fu’s *F*_s_ [42], Ewens-Watterson test, and Chakraborty’s test were calculated using Arlequin version 3.5.2.2 [31]. Ramos-Onsins and Rozas’s *R*_2_, [43] which has greater statistical power for small sample sizes, was calculated using DnaSP version 6 [30]. Significance of the differences among these values was determined using 10,000 coalescent simulations in accordance with the recommended software parameters. The mismatch distribution approach was used, in which an observed frequency distribution of pairwise nucleotide differences obtained among individuals with expected distributions form an expanding population (small raggedness index) or a stationary population (large raggedness index), in order to test for genetic signatures of historical population expansion within Thai dairy cattle populations [44,45]. These models were applied to estimate the parameters of population expansion using a generalized least-squares approach and to compute their confidence intervals by bootstrapping (10,000 replicates) as implemented in DnaSP version 6 [30]. Bayesian coalescent-based methods were then performed to evaluate the historical demographic fluctuations using the Extended Bayesian Skyline Plot (EBSP) method implemented in BEAUTi version 2.0.2 (part of the BEAST version 2.0.2 package) [46,47] by applying the HKY model, Strict Clock, and Coalescent Bayesian Skyline Model with a Gamma distribution prior. For the mean substitution rate, the prior was set as a lognormal distribution with mean of 0.626%/million years and a standard deviation of 0.516%/million years to match the rate estimated from fossil data [48,49]. TRACER was applied to assess burn-in and the effective sample sizes (ESS) of the parameters. The EBSP can fit different demographic scenarios by allowing changes in population size over time.

### 2.4. Haplogroup Classification

To investigate the haplogroups of the Thai dairy cattle population, multiple sequence alignment of 179 dairy cattle mt D-loop sequences was performed with the seven representative reference sequence as world cattle haplogroups using MEGAX [28]. Representative sequences of mtDNA haplotypes T1 (GenBank accession numbers: LC013968), T2 (AB117049), T3 (V00654), T4 (LC013966), P (DQ124389), I1 (Bhutanese native cattle: AB268579), and I2 (AB268559) were used to construct the phylogenetic trees. An unrooted neighbor-joining phylogenetic tree was constructed using the Tamura–Nei distance implemented in MEGAX [50,51].

## 3. Results

### 3.1. Genetic Diversity and Population Structure

**Figure 1:**
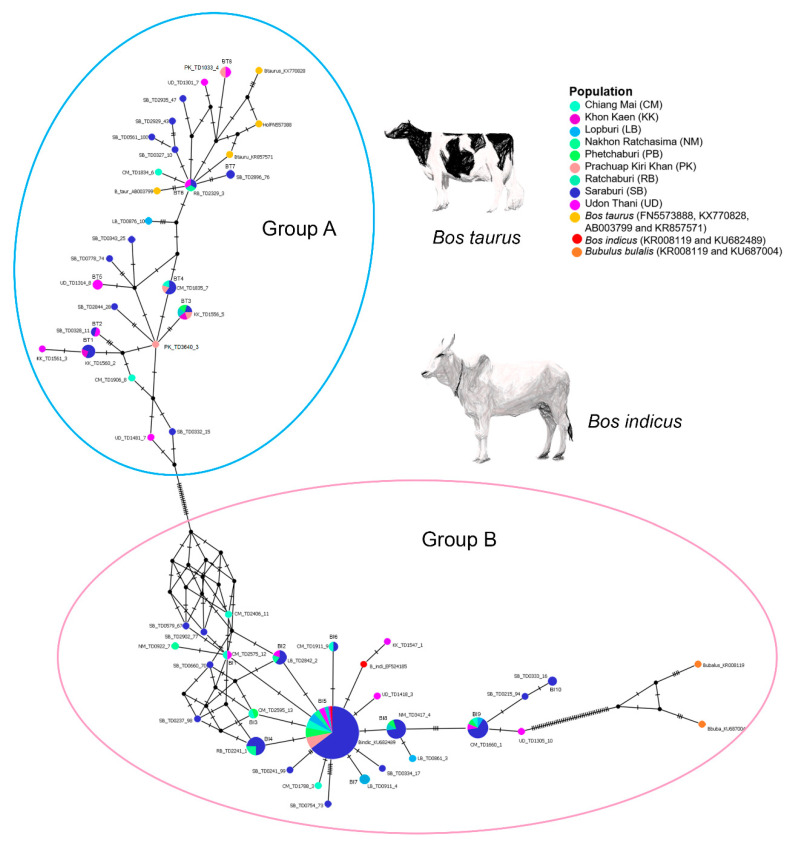
Haplotype network derived from nucleotide sequence data for mitochondrial D-loop of cattle (*Bos taurus*) for 187 (179 + 8) individuals. Different colors are used to distinguish the nine populations sampled. Each circle represents a unique DNA sequence (haplotype). Presumably the diameter of each circle reflects the total number of individuals possessing the haplotype. The number of individuals possessing each haplotype are indicated by different colors within the circles.

The amplicon length and alignment length of the mt D-loop sequences were 1200 and 723 bps, respectively. Fifty-one haplotypes were resolved from the mt D-loop sequences. The overall haplotype and nucleotide diversities were 0.791 ± 0.032 and 0.245 ± 0.012 for the mt D-loop sequences (Table 2). A complex haplotype network was constructed from the large number of detected polymorphic sites and haplotypes, and showed a strikingly star-shaped topology with two major groups (A and B) (Figure 1). The most common haplotypes (BT3) of the group A were detected in nine populations. Eight haplotypes (BT1, BT2, BT3, BT4, BT5, BT6, BT7, and BT8) were shared among the Chiang Mai, Khon Kaen, Nakhon Ratchasima, Phetchaburi, Prachuap Khiri Khan, Saraburi, or Udon Thani populations. The most common haplotype (BI5) of the group B was detected in nine populations. Ten haplotypes (BI1, BI2, BI3, BI4, BI5, BI6, BI7, BI8, BI9, and BI10) were shared among the Chiang Mai, Khon Kaen, Lopburi, Nakhon Ratchasima, Phetchaburi, Prachuap Khiri Khan, Ratchaburi, Saraburi, or Udon Thani populations.

**Figure 2:**
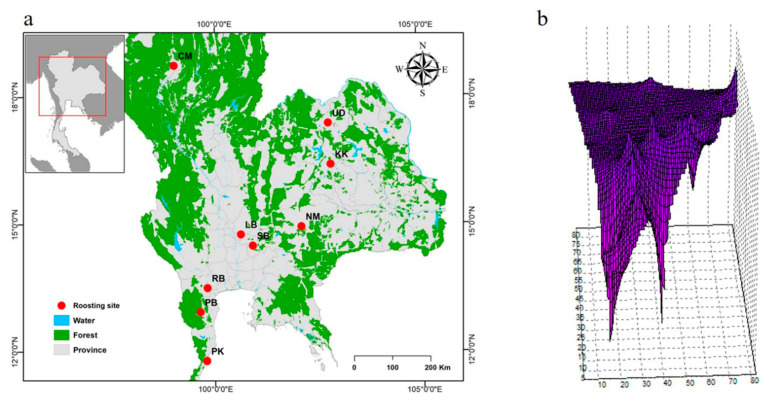
Geographic analyses of the *Bos taurus* data set. (**a**) Geographic distribution of the nine *B. taurus* populations (Chiang Mai: CM; Khon Kaen: KK; Lopburi (LB); Nakhon Ratchasima: NM; Phetchaburi: PB; Prachuap Kiri Khan: PK; Ratchaburi: RB; Saraburi: SB; and Udon Thani: UD). (**b**) Genetic landscape interpolation plots for mitochondrial D-loop sequence data depicting areas with high or low genetic differentiation based on geographic coordinates. Bright purple indicates low genetic diversities. Low nucleotide diversities within populations located on livestock farms were found in the central region (Lopburi, Phetchaburi, and Ratchaburi).

To examine the genetic differentiation among the nine populations, we calculated *F*_ST_, *G*_ST_, *Φ*_ST_, *D*_xy_, *D*_a_, and *N*_m_. The *F*_ST_ values ranged from −0.070 to 0.283, the *G*_ST_ values ranged from −0.033 and 0.044, and the *Φ*_ST_ values ranged from 0.002 to 0.257 for the mt D-loop sequences (Table 3). The *N*_m_ values ranged from 1.768 to ∞, the *D*_xy_ values ranged from 0.019 to 0.050, and the *D*_a_ values ranged from −0.004 to 0.014 for the mt D-loop sequences (Table 3). Gene flow estimates were high among all populations. The Mantel tests revealed no correlation between nucleotide diversities and geographic distance from isolation-by-distance (Figure 2). The LSI analyses revealed genetic differences, which indicates the presence of genetically divergent areas for the nine populations assessed. The LSI plots showed relatively low nucleotide diversities for the Lopburi, Phetchaburi, and Ratchaburi populations and within populations of livestock farms located in the central region (Figure 2).

### 3.2. Demography of Nine Dairy Cattle Populations

**Figure 3:**
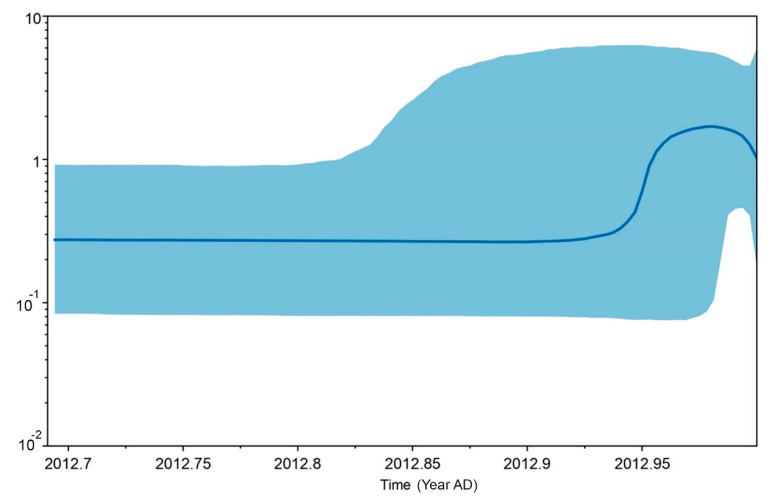
Coalescent Bayesian Skyline analysis output. The black line is the median estimated effective population size. The blue areas represent the upper and lower bounds of the 95% higher posterior density interval. The *x*-axis is time in years and the *y*-axis is a log scale.

Five different tests of neutrality were used to examine historical population expansion for mt D-loop sequences of the nine populations. The Tajima’s *D* values were not significant and ranged from −1.605 (*p* < 0.05) to 2.359 (*p* = 1.000). The Fu and Li’s *F* * values ranging from −2.149 (*p* < 0.05) to 1.673 (*p* = 1.000) were not significant. The Fu and Li’s *D* * values ranged from −1.965 (*p* < 0.05) to 1.549 (*p* = 1.000) were not significant. The Ramos-Onsins and Rozas’s *R*_2_ values ranged from 0.081 to 0.362 (Table 4). Mismatch distribution analysis indicated a multimodal distribution. The raggedness index values ranged from 0.040 to 0.240. The EBSPs based on the mt D-loop sequences detected a slight population expansion event for the Thai dairy cattle population (Figure 3). We observed that the population size remained constant over a long period with an expansion around 2012 (Figure 3).

## 4. Discussion

The process of natural selection and domestication of cattle from their wild ancestral species, in conjunction with isolation and genetic drift, has created numerous *B. taurus* breeds and broad phenotypic and genetic variation [52,53]. Intensive artificial selection has resulted in highly productive global dairy cattle breeds that have replaced local breeds, leading to a loss of diversity. Cattle show the highest number of breeds at risk of extinction among livestock [54]. Thailand has a large dairy cattle population, which is leading for milk production efficiency among ASEAN countries [55,56]. Since Thailand started commercial dairy farming promotion in 1962, the dairy cattle population and volume of milk yield have increased year-on-year. Thai dairy cattle derived from a crossbreed between *B. taurus* and *B. indicus* have adapted over 60 years to the harsh native environment, accrued resistance to tropical diseases and external parasites, and obtained sustenance on low-quality roughage and grasses following traditional methods of animal husbandry [57]. Despite the importance of dairy cattle in Thai agriculture, few attempts have been made to evaluate genetic diversity and relationships among Thai dairy cattle [56]. However, comprehensive knowledge of the genetic diversity of Thai dairy cattle, including within and between populations as an indicator of biological diversity, is required to facilitate effective management. Better knowledge of the genetic diversity and population structure of Thai dairy cattle will therefore provide a rational basis for breeding strategies and the possible use of native breeds as genetic resources to meet potential future needs for adaptation to changes in environment or production [22]. Analysis of ancient DNA indicates that Thai domestic cattle originated from China and were introduced to central Thailand between 3550 and 1700 years ago. *Bos taurus* did not spread to the central plain until at least 1500 BC [58]. This finding agrees with historical records of human immigration among countries or regions [59]. Generally, most *B. taurus* mt D-loop sequences were classified into the macro-haplogroup T, which comprises six minor haplogroups: T (originated from a center in the Southeast Asia), T1 (originated from a center in Africa), T2 (originated from a center in the Southeast Asia), T3 (originated from a center in the Southeast Asia and Europe, and also distributed in Asia), T4 (originated from a center in East Asia), and T5 (originated from a center in Italy) [24,60,61]. By contrast, all *B. indicus* mt D-loop sequences were clustered within macro-haplogroup I, which was further divided into two minor haplogroups: I1 (originating from a center in the Indus Valley) and I2 (originating from a center in northern India) [24]. In this study, most mt D-loop sequences of Thai dairy cattle were classified into haplogroups T3, I1, and I2, and were members of the same haplogroup as cattle from Asia, Europe, and Africa [24]. A total of 22.35% of Thai dairy cattle were resolved into the same clade distributed in a minor haplogroup (T3) as the maternal lineage of *B. taurus*, whereas 77.65% of samples were only detected in cattle from India (haplogroups I1 and I2) as the maternal lineage of *B. indicus*. Similarly, ancient cattle haplogroups originated in Southeast, South, and Southwest China and/or surrounding areas (i.e., Vietnam, Burma, Thailand, and India), among which *B. taurus* is likely to be classified as haplogroup T3 [62]. Accounts of immigration consistently state that members of a family moved together with their cattle, which may have resulted in the introduction of ancient cattle from eastern Asia into Thailand [58]. The majority of Thai dairy cattle sampled in the present study were similar to ancient cattle maternal lineages, consistent with the contention that dairy cattle in Thailand have been crossbred between various breeds of *B. indicus* (such as Red Sindhi, Sahiwal, Brahman, and Thai native) and *B. taurus* (such as Brown Swiss, Jersey, and Red Danish) [63]. However, no specific haplogroup distribution was found in nine provinces examined. This might result from expansion of the dairy cattle business by human-mediated distribution.

In 2019, the number of dairy cattle in Thailand was 666,311, of which 46% were milking cows that produced 2000 tons of milk per day [13]. Efforts to increase milk production in Thailand combined with government policies resulted in widespread import and use of Holstein semen, and extensive use of high-percentage Holstein sires raised in Thailand by the DPO and the Department of Livestock Development [16]. This mating strategy created the Thai multi-breed dairy population, through human-mediated and natural selection, which is adapted to different agroclimatic and sociocultural conditions in Thailand [16]. We hypothesized that Thai dairy cattle achieved genetic connectivity throughout the population, even in farms within the central area, through transportation. The maternal lineage of Thai dairy cattle, analyzed using mt D-loop sequences, showed low nucleotide diversity (π) but high haplotype diversity (*h*). Commonly, the π value offers a more reliable reflection of mtDNA diversity in a population than the *h* value [29], which reflects recent changes in a population [64]. This result was also consistent with the non-significant raggedness values, which indicates that recent population expansion has occurred, resulting in turn in a complex network with a star-shaped topology [65,66]. Mismatch distribution plots showed a multimodal and ragged shape in all populations, which suggests demographic equilibrium or population stability, as also revealed by the results of neutrality tests [67]. Thus, Thai dairy cattle populations may have arisen by rapid growth with high gene flow. Population size probably increased during 2012 with increased dairy semen distribution in Thailand [68]. Simultaneously, overall neutrality test statistics in most populations were not statistically significant, with a population equilibrium that resulted from the influence of high gene flow [69]. Most *F*_ST_ values for population pairs were less than 0.25, which suggests that genetic differentiation did not occur between populations without isolation-by-distance. Average *G*_ST_ and *Φ*_ST_ values were −0.003 and 0.056, respectively, which indicates that genetic diversity occurred predominantly within populations. The relationship of *D*_xy_ and *D*_a_ values with *N*_m_ values revealed that the Thai dairy cattle populations showed historical divergence but gene flow frequently occurred between populations. This result suggests that high gene flow between populations reduced or prevented genetic differentiation. However, the limited number of samples analyzed must be noted [70]. Spatial genetic patterns in the Thai dairy cattle population, as revealed by LSI analysis, suggest that provinces differed in their degree of nucleotide diversity. We observed low nucleotide diversity within the populations of Nakhon Ratchasima, Saraburi, Prachuap Kiri Khan, and Ratchaburi. The provinces of Saraburi and Nakhon Ratchasima operated a school milk program implemented by the DPO since 2009 [18], and the dairy cattle population has subsequently increased under the promotion and extension of government projects and policies [71]. Most dairy cattle farms in Saraburi and Nakhon Ratchasima exchanged dairy cattle to improve their milk production, due to the convenience of transportation and government policy to promote using purebred and crossbred Holstein frozen semen for mating dairy cows of the Thai farmers throughout the country. The maternal lineage of Thai dairy cattle in Lopburi, Phetchaburi, Saraburi, and Ratchaburi formed a distinct cluster. This finding reflects the possible low nucleotide diversity in these populations. Interestingly, genetic trends in the dairy cattle population, generated by crossing Holstein cattle with other breeds in central Thailand from 1991 to 2005, were small for milk yield, and almost zero for fat yield and fat proportion [5], which may reflect inbreeding from some high-percentage Holstein sires. Apart from the Nakhon Ratchasima, Saraburi, Prachuap Kiri Khan, and Ratchaburi populations, the genetic variability of the remaining populations was higher because dairy farmers bought various female cattle from outside the population to start and expand their dairy business, and then they were bred with sires. Thus, these cattle were domesticated from a larger genetic pool, and the breeding tract appears to be the most likely candidate.

The network analysis reveals two major nodes, with an indication of expansion of the founding haplotypes, in an otherwise complex network derived from several singletons. The two major nodes (A and B) were connected by 17 mutational steps to the remaining nodes. Approximately 37.5% of the sampled individuals from Saraburi belonged to the major node A and 62.5% to the major node B group (Table S1). This result suggests that most dairy cattle populations were an outcome of gene flow from *B. indicus* in the recent past. By contrast, individual contributions of the remaining 73 populations ranged from 14.84% in the case of Udon Thani, Chiangmai, Prachuap Khiri Khan, Khon Kaen, and Lopburi populations in the A clade, to 59.36% in the Chiang Mai, Nakhon Ratchasima, Khon Kaen, Ratchaburi, Lopburi, Udon Thani, and Phetchaburi populations in the B clade. This finding points to a decline in D-loop haplotype complexity in these populations with distance from the sampling sites in the Saraburi population. This may be the result of intensive selection operating mainly through paternal lineages. However, it was surprising to note a strong effect on mitochondrial genetic diversity. Certain branches in the network are indicative of ancient differentiation of some maternal lineages of *B. taurus* and *B. indicus*. These results have important implications for rational decision-making involved with breeding strategy and observations of breeding value. Availability of whole-genome single-nucleotide polymorphism arrays has improved the accuracy of genomic studies and provided information on the current status of cattle genetic resources [72].

## 5. Conclusions

The modernization of agriculture, the current high intensity of economic competition, subdivision of land holdings, introduction of highly productive breeds, and demographic pressure all contribute to loss of valuable traits or decreases in the populations of local breeds. Farmers must increase the efficiency of high-quality milk production and reduce costs to improve their profitability. To identify factors that affect milk production and revenue, and evaluate their economic importance [73,74], it is first necessary to manage pedigrees to reduce the degree of homozygosity and inbreeding in Thai dairy cattle. The inbreeding depression partially observed in the present study population does not affect economic traits, but needs to be overcome to maximize the long-term performance and health of cattle populations [75]. This information would also assist dairy cooperatives and private organizations to provide more appropriate and effective support to their members. Genetic improvement is a fundamental task that is concerned primarily with the development of a high-yielding and efficient dairy cattle breed for smallholder dairy farmers [76]. Moreover, a database of experience and economic performance could provide important knowledge for organizations to plan training programs for dairy farmers [17]. Basic databases are useful to guide future herd and dairy research in Thailand. Knowledge of genetic diversity, genetic distinctiveness, and population genetic structure provides critical information for management of animal genetic resources. Novel crossbreeding technologies can be used to elucidate Thai dairy cattle traits for milk yield using genetic approaches, analysis of environmental factors, and surveys of farmer experience [12,77]. In this study, we analyzed the mt D-loop sequence variation in nine Thai dairy cattle populations, with a focus on the exploration of the origin and genetic diversity of the cattle. The present sampling did not include all individuals in the populations but was useful to provide an overview of the whole genetic pool and to trace matrilineal origins. Our analyses of mitochondrial analyses provide evidence for historical genetic connectivity between two distinct background genetic resources, namely *B. taurus* and *B. indicus*. Building on the recent strategy for agricultural production, the Thai Government must assist farmers in adopting new technologies as a means of practicing sustainable agriculture.

## Figures and Tables

**Table 1:** Sampled populations of cattle (*Bos taurus*) in nine provinces of Thailand.

No.	Abbreviation/Code	Province	Geographic Coordinates	Number of Cattle Samples	Mitochondrial D-Loop GenBank Accession Number
1	CM	Chiang Mai	18°47′15″ N, 98°59′35″ E	13	LC604297–LC604309
2	KK	Khon Kaen	16°26′30″ N, 102°50′9″ E	5	LC604310–LC604314
3	LB	Lopburi	14°48′00″ N, 100°37′37″ E	10	LC604315–LC604324
4	NM	Nakhon Ratchasima	14°58′50″ N, 102°06′00″ E	8	LC604325–LC604332
5	PB	Phetchaburi	12°57′53″ N, 99°38′33″ E	6	LC604333–LC604338
6	PK	Prachuap Kiri Khan	11°48′44″ N, 99°47′50″ E	11	LC604339–LC604349
7	RB	Ratchaburi	13°32′08″ N, 99°48′48″ E	6	LC604350–LC604355
8	SB	Saraburi	14°31′59″ N, 100°55′00″ E	106	LC604356–LC604461
9	UD	Udon Thani	17°24′49″ N, 102°47′14″ E	14	LC604462–LC604475

**Table 2:** Mitochondrial D-loop sequence diversity for cattle in all sampled populations of Thailand.

Population	Sample Size	Number of Haplotypes (*H*)	Haplotype Diversity (*h*)	Nucleotide Diversity (*π*)	Theta (Per Site; *S*)	Average Number of Nucleotide Differences (*k*)	Haploguops
Chiang Mai (CM)	13	10	0.923 ± 0.069	0.202 ± 0.104	0.021	14.795	I1, I2, T3
Khon Kaen (KK)	5	5	1.000 ± 0.126	0.188 ± 0.114	0.026	22.000	I1, T3
Lopburi (LB)	10	6	0.844 ± 0.103	0.247 ± 0.131	0.033	7.978	I1, I2, T3
Nakhon Ratchasima (NM)	8	4	0.643 ± 0.184	0.223 ± 0.122	0.020	9.179	I1, T3
Phetchaburi (PB)	6	6	1.000 ± 0.096	0.228 ± 0.132	0.024	13.467	I1, I2, T3
Prachuap Kiri Khan (PK)	11	7	0.818 ± 0.119	0.237 ± 0.124	0.018	17.345	I1, T3
Ratchaburi (RB)	6	3	0.733 ± 0.155	0.251 ± 0.147	0.020	11.267	I1, T3
Saraburi (SB)	106	31	0.760 ± 0.044	0.214 ± 0.103	0.019	11.803	I1, I2, T3
Udon Thani (UD)	14	11	0.956 ± 0.045	0.171 ± 0.088	0.02	20.132	I1, I2, T3
All population	179	51	0.791 ± 0.032	0.245 ± 0.012	0.032	11.682	I1, I2, T3

**Table 3:** Genetic differentiation between the nine populations in Thailand of cattle based on mitochondrial D-loop sequences. *G*_ST_, genetic differentiation coefficient; *F*_ST_, Wright’s *F*-statistic for subpopulations within the total population; *Φ*_ST_; *N*_m_, gene flow from sequence data and haplotype data; *D*_xy_, average number of nucleotide substitutions per site between populations; *D*_a_, net nucleotide substitutions per site between populations.

Population 1	Population 2	*G*_ST_	*Φ*_ST_	*F*_ST_	*D*_xy_	*D*_a_	*N*_m_
CM	KK	0.004	0.124	0.114	0.047	0.005	∞ ^1^
CM	LB	−0.007	0.031	−0.025	0.026	−0.001	∞
CM	NM	0.017	0.023	−0.050	0.026	−0.001	5.519
CM	PB	−0.003	0.014	−0.098	0.029	−0.003	∞
CM	PK	0	0.022	−0.046	0.035	−0.002	21.06
CM	RB	0.017	0.018	−0.083	0.027	−0.002	∞
CM	SB	0.015	0.003	−0.034	0.029	−0.001	19.325
CM	UD	−0.006	0.066	0.055	0.042	0.002	∞
KK	LB	0.007	0.257	0.283	0.05	0.014	8.438
KK	NM	0.021	0.244	0.25	0.048	0.012	2.224
KK	PB	−0.033	0.189	0.157	0.049	0.008	∞
KK	PK	0.015	0.065	−0.018	0.044	−0.001	6.301
KK	RB	0.02	0.219	0.205	0.048	0.01	25.794
KK	SB	0.044	0.045	0.2	0.049	0.01	4.937
KK	UD	0.007	0.028	−0.081	0.045	−0.004	∞
LB	NM	0.005	0.015	−0.093	0.019	−0.002	∞
LB	PB	0.001	0.022	−0.111	0.023	−0.003	∞
LB	PK	−0.008	0.083	0.068	0.032	0.002	∞
LB	RB	0.011	0.025	−0.099	0.021	−0.002	∞
LB	SB	0.018	0.004	−0.022	0.023	−0.001	∞
LB	UD	0.005	0.144	0.205	0.042	0.009	4.659
NM	PB	0.021	0.019	−0.133	0.023	−0.003	114.157
NM	PK	−0.026	0.068	0.038	0.031	0.001	∞
NM	RB	0.001	0.028	−0.111	0.021	−0.002	∞
NM	SB	0.022	0.003	−0.038	0.023	−0.001	∞
NM	UD	0.039	0.127	0.187	0.041	0.008	1.768
PB	PK	0.022	0.042	−0.037	0.034	−0.001	∞
PB	RB	0.028	0.033	−0.124	0.025	−0.003	∞
PB	SB	0.037	0.002	−0.082	0.027	−0.002	∞
PB	UD	−0.002	0.079	0.083	0.042	0.003	∞
PK	RB	0.003	0.049	−0.015	0.032	0	∞
PK	SB	0.013	0.016	0.019	0.034	0.001	∞
PK	UD	0.02	0.023	−0.038	0.041	−0.002	3.595
RB	SB	0.033	0.002	−0.070	0.025	−0.002	∞
RB	UD	0.026	0.091	0.119	0.041	0.005	6.954
SB	UD	0.022	0.051	0.132	0.042	0.006	3.787

^1^ Presence of high gene flow.

**Table 4:** Neutrality tests of mitochondrial D-loop sequences from nine cattle populations in Thailand.

Population	Tajima’s *D*	Fu and Li’s D *	Fu and Li’s F *	Fu’s *F*_s_	Ewens-Watterson Test	Chakraborty’s Test	Ramos-Onsins and Rozas	Raggedness Index
Chiang Mai (CM)	0.552 ^ns^	0.241 ^ns^	0.170 ^ns^	0.996 ^ns^	1.000 ^ns^	0.327 ^ns^	0.14	0.040 ^ns^
Khon Kaen (KK)	2.359 ^ns^	1.549 ^ns^	1.673 ^ns^	0.565 ^ns^	N.A.	N.A.	0.269	0.200 ^ns^
Lopburi (LB)	−1.605 *	−1.919 ^ns^	−2.134 ^ns^	1.000 ^ns^	0.903 ^ns^	0.525 ^ns^	0.242	0.105 ^ns^
Nakhon Ratchasima (NM)	−1.541 *	−1.965 *	−2.149 *	1.000 ^ns^	1.000 ^ns^	0.351 ^ns^	0.291	0.166 ^ns^
Phetchaburi (PB)	−0.701 ^ns^	−1.216 ^ns^	−1.331 ^ns^	0.963 ^ns^	1.000 ^ns^	0.766 ^ns^	0.289	0.053 ^ns^
Prachuap Kiri Khan (PK)	1.737 ^ns^	1.048 ^ns^	1.343 ^ns^	1.000 ^ns^	1.000 ^ns^	0.184 ^ns^	0.218	0.191 ^ns^
Ratchaburi (RB)	−0.939 ^ns^	−1.458 ^ns^	−1.581 ^ns^	1.000 ^ns^	0.611 ^ns^	0.756 ^ns^	0.362	0.240 ^ns^
Saraburi (SB)	−0.088 ^ns^	−1.413 ^ns^	−1.196 ^ns^	1.000 ^ns^	1.000 ^ns^	0.000 **	0.081	0.043 ^ns^
Udon Thani (UD)	2.077 ^ns^	0.999 ^ns^	1.413 ^ns^	0.977 ^ns^	0.938 ^ns^	0.605 ^ns^	0.215	0.045 ^ns^
All populations	−0.232 ^ns^	−2.537 *	−1.768 ^ns^	−2.530 ^ns^	1.000 ^ns^	0.000 **	0.14	0.022 ^ns^

Significant differentiation values ** *p* < 0.01, * *p* < 0.05; ns = not significant value.

## Data Availability

Nucleotide sequence data generated in this study were deposited in DNA Data Bank of Japan (DDBJ) (accession number LC604297–LC604475).

## Supplementary Materials

The following are available online at https://www.mdpi.com/article/10.3390/ani11061680/s1, Table S1: Sampled populations and haplogroup of cattle at nine provinces in Thailand. All sequences were deposited in the DNA Data Bank of Japan (DDBJ).
