# Supplementary material for: High-Level Gene Flow Restricts Genetic Differentiation in Dairy Cattle Populations in Thailand: Insights from Large-Scale Mt D-Loop Sequencing
Source: Animals (Basel). 2021 Jun 4;11(6):1680. doi: 10.3390/ani11061680 (PMC8227385; doi:10.3390/ani11061680)
Supplement: Supplementary file 1 [file animals-11-01680-s001.zip › animals-1195854-supplementary.pdf]

Table S1. Sampled populations and haplogroup of cattle at nine provinces in Thailand. All sequences were deposited in the DNA Data Bank of Japan (DDBJ).

No.	Abbreviation/ Code	Province	Mitochondrial D-Loop GenBank Accession Number	Haplogroup	Group[1]
1	CM_TD1660_1	Chiang Mai	LC604297	I2	B[2]
2	CM_TD1779_2	Chiang Mai	LC604298	I1	B
3	CM_TD1788_3	Chiang Mai	LC604299	I1	B
4	CM_TD1832_4	Chiang Mai	LC604300	I1	B
5	CM_TD1833_5	Chiang Mai	LC604301	I1	B
6	CM_TD1834_6	Chiang Mai	LC604302	I1	B
7	CM_TD1835_7	Chiang Mai	LC604303	T3	A[3]
8	CM_TD1906_8	Chiang Mai	LC604304	T3	A
9	CM_TD1911_9	Chiang Mai	LC604305	I1	B
10	CM_TD1922_10	Chiang Mai	LC604306	I1	B
11	CM_TD2406_11	Chiang Mai	LC604307	I1	B
12	CM_TD2575_12	Chiang Mai	LC604308	I1	B
13	CM_TD2595_13	Chiang Mai	LC604309	I1	B
14	KK_TD1544_4	Khon Kaen	LC604310	I1	B
15	KK_TD1547_1	Khon Kaen	LC604311	I1	B
16	KK_TD1556_5	Khon Kaen	LC604312	T3	A
17	KK_TD1560_2	Khon Kaen	LC604313	I1	B
18	KK_TD1561_3	Khon Kaen	LC604314	T3	A
19	LB_TD0856_7	Lopburi	LC604315	I1	B
20	LB_TD0858_8	Lopburi	LC604316	I1	B
21	LB_TD0861_3	Lopburi	LC604317	I1	B
22	LB_TD0876_10	Lopburi	LC604318	T3	A
23	LB_TD0876_9	Lopburi	LC604319	I1	B
24	LB_TD0911_4	Lopburi	LC604320	I1	B
25	LB_TD0912_5	Lopburi	LC604321	I1	B
26	LB_TD0913_6	Lopburi	LC604322	I1	B
27	LB_TD2789_1	Lopburi	LC604323	I2	B
28	LB_TD2842_2	Lopburi	LC604324	T3	A
29	NM_TD0922_7	Nakhon Ratchasima	LC604325	I1	B
30	NM_TD3017_1	Nakhon Ratchasima	LC604326	I1	B
31	NM_TD3019_2	Nakhon Ratchasima	LC604327	I1	B
32	NM_TD3045_8	Nakhon Ratchasima	LC604328	I1	B
33	NM_TD3253_3	Nakhon Ratchasima	LC604329	T3	A
34	NM_TD3417_4	Nakhon Ratchasima	LC604330	I1	B
35	NM_TD3488_5	Nakhon Ratchasima	LC604331	I1	B
36	NM_TD3509_6	Nakhon Ratchasima	LC604332	I1	B
37	PB_TD1050_1	Phetchaburi	LC604333	I1	B
38	PB_TD1079_2	Phetchaburi	LC604334	I2	B
39	PB_TD1138_6	Phetchaburi	LC604335	I1	B
40	PB_TD1139_3	Phetchaburi	LC604336	T3	A
41	PB_TD1157_4	Phetchaburi	LC604337	I1	B
42	PB_TD1176_5	Phetchaburi	LC604338	I1	B

No.	Abbreviation/ Code	Province	Mitochondrial D-loop GenBank accession number	Haplogroup	Group
43	PK_TD1001_11	Prachuap Kiri Khan	LC604339	I1	B
44	PK_TD1002_9	Prachuap Kiri Khan	LC604340	I1	B
45	PK_TD1033_4	Prachuap Kiri Khan	LC604341	T3	A
46	PK_TD1066_5	Prachuap Kiri Khan	LC604342	I1	B
47	PK_TD1202_10	Prachuap Kiri Khan	LC604343	I1	B
48	PK_TD1210_6	Prachuap Kiri Khan	LC604344	T3	A
49	PK_TD1242_7	Prachuap Kiri Khan	LC604345	I1	B
50	PK_TD1276_8	Prachuap Kiri Khan	LC604346	T3	A
51	PK_TD3629_1	Prachuap Kiri Khan	LC604347	I1	B
52	PK_TD3630_2	Prachuap Kiri Khan	LC604348	I1	B
53	PK_TD3640_3	Prachuap Kiri Khan	LC604349	I1	B
54	RB_TD2241_1	Ratchaburi	LC604350	I1	B
55	RB_TD2249_6	Ratchaburi	LC604351	I1	B
56	RB_TD2264_2	Ratchaburi	LC604352	I1	B
57	RB_TD2329_3	Ratchaburi	LC604353	T3	A
58	RB_TD2335_4	Ratchaburi	LC604354	I1	B
59	RB_TD2339_5	Ratchaburi	LC604355	I1	B
60	SB_TD0048_1	Saraburi	LC604356	I1	B
61	SB_TD0062_78	Saraburi	LC604357	I1	B
62	SB_TD0080_79	Saraburi	LC604358	I1	B
63	SB_TD0103_80	Saraburi	LC604359	I1	B
64	SB_TD0111_81	Saraburi	LC604360	I1	B
65	SB_TD0132_82	Saraburi	LC604361	I1	B
66	SB_TD0140_83	Saraburi	LC604362	I1	B
67	SB_TD0147_84	Saraburi	LC604363	I1	B
68	SB_TD0154_85	Saraburi	LC604364	I1	B
69	SB_TD0162_86	Saraburi	LC604365	I1	B
70	SB_TD0165_87	Saraburi	LC604366	I1	B
71	SB_TD0186_88	Saraburi	LC604367	I1	B
72	SB_TD0194_90	Saraburi	LC604368	I1	B
73	SB_TD0204_91	Saraburi	LC604369	I2	B
74	SB_TD0206_89	Saraburi	LC604370	I1	B
75	SB_TD0209_92	Saraburi	LC604371	I1	B
76	SB_TD0211_93	Saraburi	LC604372	I1	B
77	SB_TD0215_94	Saraburi	LC604373	I2	B
78	SB_TD0217_95	Saraburi	LC604374	I1	B
79	SB_TD0228_96	Saraburi	LC604375	I1	B
80	SB_TD0235_97	Saraburi	LC604376	I1	B
81	SB_TD0237_98	Saraburi	LC604377	I1	B
82	SB_TD0241_99	Saraburi	LC604378	I1	B
83	SB_TD0255_101	Saraburi	LC604379	I1	B
84	SB_TD0318_2	Saraburi	LC604380	I2#	B
85	SB_TD0319_3	Saraburi	LC604381	I1	B
86	SB_TD0320_4	Saraburi	LC604382	I1	B
87	SB_TD0321_5	Saraburi	LC604383	I1	B
88	SB_TD0322_6	Saraburi	LC604384	I2	B
89	SB_TD0323_7	Saraburi	LC604385	I1	B

No.	Abbreviation/ Code	Province	Mitochondrial D-loop GenBank accession number	Haplogroup	Group
90	SB_TD0324_8	Saraburi	LC604386	I1	B
91	SB_TD0327_9	Saraburi	LC604387	I2	B
92	SB_TD0327_10	Saraburi	LC604388	T3	A
93	SB_TD0328_11	Saraburi	LC604389	T3	A
94	SB_TD0329_12	Saraburi	LC604390	I1	B
95	SB_TD0330_13	Saraburi	LC604391	I2	B
96	SB_TD0331_14	Saraburi	LC604392	I1	B
97	SB_TD0332_15	Saraburi	LC604393	T3	A
98	SB_TD0333_16	Saraburi	LC604394	I2	B
99	SB_TD0334_17	Saraburi	LC604395	I1	B
100	SB_TD0335_18	Saraburi	LC604396	I2	B
101	SB_TD0336_19	Saraburi	LC604397	T3	A
102	SB_TD0337_20	Saraburi	LC604398	I2	B
103	SB_TD0338_21	Saraburi	LC604399	I1	B
104	SB_TD0339_22	Saraburi	LC604400	I1	B
105	SB_TD0341_23	Saraburi	LC604401	I1	B
106	SB_TD0342_24	Saraburi	LC604402	I1	B
107	SB_TD0343_25	Saraburi	LC604403	T3	A
108	SB_TD0561_100	Saraburi	LC604404	T3	A
109	SB_TD0579_67	Saraburi	LC604405	I1	B
110	SB_TD0629_65	Saraburi	LC604406	I1	B
111	SB_TD0656_68	Saraburi	LC604407	I1	B
112	SB_TD0659_69	Saraburi	LC604408	T3	A
113	SB_TD0660_70	Saraburi	LC604409	I1	B
114	SB_TD0662_71	Saraburi	LC604410	I1	B
115	SB_TD0728_72	Saraburi	LC604411	I1	B
116	SB_TD0754_73	Saraburi	LC604412	I1	B
117	SB_TD0778_74	Saraburi	LC604413	T3	A
118	SB_TD0895_75	Saraburi	LC604414	I1	B
119	SB_TD2050_26	Saraburi	LC604415	I1	B
120	SB_TD2099_27	Saraburi	LC604416	T3	A
121	SB_TD2844_28	Saraburi	LC604417	T3	A
122	SB_TD2845_29	Saraburi	LC604418	I1	B
123	SB_TD2847_30	Saraburi	LC604419	I1	B
124	SB_TD2855_31	Saraburi	LC604420	I1	B
125	SB_TD2858_32	Saraburi	LC604421	I1	B
126	SB_TD2861_33	Saraburi	LC604422	I1	B
127	SB_TD2863_34	Saraburi	LC604423	I1	B
128	SB_TD2865_35	Saraburi	LC604424	I1	B
129	SB_TD2879_36	Saraburi	LC604425	I1	B
130	SB_TD2896_76	Saraburi	LC604426	T3	A
131	SB_TD2898_37	Saraburi	LC604427	I1	B
132	SB_TD2900_63	Saraburi	LC604428	I1	B
133	SB_TD2902_77	Saraburi	LC604429	I1	B
134	SB_TD2903_66	Saraburi	LC604430	I1	B
135	SB_TD2904_64	Saraburi	LC604431	T3	A
136	SB_TD2916_38	Saraburi	LC604432	T3	A

No.	Abbreviation/ Code	Province	Mitochondrial D-loop GenBank accession number	Haplogroup	Group
137	SB_TD2924_39	Saraburi	LC604433	I2	B
138	SB_TD2925_40	Saraburi	LC604434	I1	B
139	SB_TD2926_41	Saraburi	LC604435	I1	B
140	SB_TD2928_42	Saraburi	LC604436	T3	A
141	SB_TD2929_43	Saraburi	LC604437	T3	A
142	SB_TD2930_44♯	Saraburi	LC604438	T3	A♯
143	SB_TD2932_45	Saraburi	LC604439	I1	B
144	SB_TD2933_46	Saraburi	LC604440	I1	B
145	SB_TD2935_47	Saraburi	LC604441	T3	A
146	SB_TD2936_48	Saraburi	LC604442	I1	B
147	SB_TD2943_49	Saraburi	LC604443	I1	B
148	SB_TD2944_50	Saraburi	LC604444	I1	B
149	SB_TD2945_51	Saraburi	LC604445	I1	B
150	SB_TD2948_52	Saraburi	LC604446	I1	B
151	SB_TD3599_53	Saraburi	LC604447	I1	B
152	SB_TD3600_54	Saraburi	LC604448	T3	A
153	SB_TD3601_55	Saraburi	LC604449	I1	B
154	SB_TD3602_56	Saraburi	LC604450	I1	B
155	SB_TD3603_57	Saraburi	LC604451	I1	B
156	SB_TD3604_58	Saraburi	LC604452	I1	B
157	SB_TD3605_102	Saraburi	LC604453	I1	B
158	SB_TD3606_103	Saraburi	LC604454	I1	B
159	SB_TD3607_59	Saraburi	LC604455	I1	B
160	SB_TD3612_60	Saraburi	LC604456	T3	A
161	SB_TD3613_61	Saraburi	LC604457	I1	B
162	SB_TD3616_104	Saraburi	LC604458	I1	B
163	SB_TD3620_105	Saraburi	LC604459	I1	B
164	SB_TD3622_106	Saraburi	LC604460	I1	B
165	SB_TD3623_62	Saraburi	LC604461	I1	B
166	UD_TD1301_7	Udon Thani	LC604462	T3	A
167	UD_TD1305_10	Udon Thani	LC604463	I2	B
168	UD_TD1314_8	Udon Thani	LC604464	T3	A
169	UD_TD1316_13	Udon Thani	LC604465	T3	A
170	UD_TD1341_11	Udon Thani	LC604466	I2	B
171	UD_TD1357_12	Udon Thani	LC604467	I1	B
172	UD_TD1363_9	Udon Thani	LC604468	T3	A
173	UD_TD1392_1	Udon Thani	LC604469	T3	A
174	UD_TD1400_2	Udon Thani	LC604470	T3	A
175	UD_TD1418_3	Udon Thani	LC604471	I1	B
176	UD_TD1428_4	Udon Thani	LC604472	I1	B
177	UD_TD1441_5	Udon Thani	LC604473	I1	B
178	UD_TD1443_6	Udon Thani	LC604474	I1	B
179	UD_TD1481_14	Udon Thani	LC604475	I1	B

[1]Major group characterized by statistical parsimony network of consensus sequences (Figure 1).
[2]Reference nucleotide sequence of *Bos indicus* (Gen Bank accession number: EF524185 and KU682489).
[3]Reference nucleotide sequence of *Bos tuarus* (Gen Bank accession number: FN5573888, KX770828, AB003799, and KR857571).
